# Management of lateral skull base cancer: United Kingdom National Multidisciplinary Guidelines

**DOI:** 10.1017/S0022215116000542

**Published:** 2016-05

**Authors:** JJ Homer, T Lesser, D Moffat, N Slevin, R Price, T Blackburn

**Affiliations:** 1 Manchester Academic Health Sciences Centre, University of Manchester, Manchester, UK; 2 Department of ENT/Head and Neck Surgery, University Hospital Aintree, Liverpool, UK; 3 Department of Neuro-otology and Skull Base Surgery, University of Cambridge, Cambridge University Teaching Hospitals NHS Foundation Trust, Cambridge, UK; 4 Department of Clinical Oncology, Christie Hospital NHS Trust, Manchester, UK; 5 Department of Plastic and Reconstructive Surgery, Cambridge University Teaching Hospitals NHS Foundation Trust, Cambridge, UK; 6 Department of Oral and Maxillofacial Surgery, Manchester Royal Infirmary, Manchester, UK

## Abstract

This is the official guideline endorsed by the specialty associations involved in the care of head and neck cancer patients in the UK. It provides recommendations on the work up and management of lateral skull base cancer based on the existing evidence base for this rare condition.

**Recommendations:**

• All patients with more than one of: chronic otalgia, bloody otorrhoea, bleeding, mass, facial swelling or palsy should be biopsied. (R)

• Magnetic resonance and computed tomography imaging should be performed. (R)

• Patients should undergo audiological assessment. (R)

• Carotid angiography is recommended in select patients. (G)

• The modified Pittsburg T-staging system is recommended. (G)

• The minimum operation for cancer involving the temporal bone is a lateral temporal bone resection. (R)

• Facial nerve rehabilitation should be initiated at primary surgery. (G)

• Anterolateral thigh free flap is the workhorse flap for lateral skull base defect reconstruction. (G)

• For patients undergoing surgery for squamous cell carcinoma, at least a superficial parotidectomy and selective neck dissection should be carried out. (R)

## Introduction

Primary cancers of the temporal bone (TB) and lateral skull base are comparatively rare, accounting for 0.2 per cent of all head and neck cancers. They consist of different sites of cancer with a range of pathologies. Consequently, there is little evidence as to best practice. Over ten times more frequent are cancers of the skin and parotid invading the TB. Despite this there is even less evidence of best practice. Lateral skull base cancer can be considered to comprise any of the entities described in Table I.

**Table I:** Entities that come under the category of lateral skull base cancer

Site	Main pathologies
Advanced skin cancer (conchal bowl/pinna/peri-auricular skin)	SCC BCC Melanoma
Advanced parotid cancers (involving ear/temporal bone)	Salivary gland malignant neoplasms (generally high grade), including metastatic skin SCC to intra-parotid lymph nodes
Infratemporal fossa temporo-mandibular joint	Sarcomas (e.g. chondrosarcomas, rhabdosarcoma, osteosarcoma)
EAM/ME	Most SCC (80%) BCC Skin adnexal cancers

## Clinical presentation

Late diagnosis of patients with cancers of the external auditory meatus (EAM) and middle ear (ME) is not uncommon and this should be considered in any patients with: chronic otalgia, bloody otorrhoea, bleeding, mass, facial swelling or palsy.^1^ Clinical findings of excoriation, ulceration and granulation tissue should be considered as suspicious. Some patients may have a long history of chronic middle or external ear infection, which can be a pre-disposing factor.

Skin cancers present as visible or itchy skin and/or pinna lesions. Tumours of the infratemporal fossa may present with a subtle mass or fullness immediately above the zygoma or with pain (which can be easily misdiagnosed as temporal mandibular joint pain).

## Assessment and staging

### Clinical examination

Confirmation of diagnosis is mandatory before treatment and is gained by biopsy of the pinna, skin, EAM or ME. Advanced parotid cancers should be diagnosed through cytopathology or, occasionally if necessary, incision biopsy. Tumours of the infratemporal fossa often will require a surgical biopsy via access superior or inferior to the zygoma as necessary. Cytology is possible, but, as many tumours here are sarcomas, histopathology is required. The differential diagnosis is myriad, but care must be taken to exclude pseudotumoral skull base osteomyelitis of the TB (also called necrotising otitis externa) and inflammatory diseases such as granulomatosis with polyangiitis.

### Imaging considerations

In most cases, both computed tomography (CT) and magnetic resonance imaging (MRI) should be used. Computed tomography (fine cut, high resolution) is essential for external auditory canal (EAC) erosion, extent of middle ear and mastoid involvement, spread into jugular bulb, carotid canal, tegmen, temporomandibular joint (TMJ), parotid and beyond. It can also stage the neck. Magnetic resonance  differentiates mucosal swelling or mastoid fluid from tumour; is superior at ascertaining dural or brain involvement; and gives more detail of parapharyngeal space and infratemporal fossa involvement.

Despite high-resolution scanning using both modalities, both over and under estimation of the extent of the tumour occurs. Patients should be prepared for more extensive surgery or abandoning surgery if the scans prove wrong.

Depending on the pathology of the tumour, imaging of the thorax (squamous cell carcinoma (SCC)) or whole body may be required (sarcomas, melanoma).

Carotid angiography and balloon occlusion are occasionally required to assess ipsilateral carotid artery involvement. If a tumour is thought unresectable without internal carotid artery sacrifice, then a temporary balloon occlusion test can be performed. If successful, permanent pre-operative occlusion via coils can be performed (ideally two weeks pre-operatively).

### Audiology

Pure tone audiogram of both ears should be performed pre-operatively.

### Pre-treatment staging

There is no Union for International Cancer Control (UICC) or American Joint Committee on Cancer (AJCC) staging system for cancers of the TB or lateral skull base. However, many use the revised Pittsburgh staging system (Table II). The standard UICC staging is used for neck and distant metastases.

**Recommendations**

• All patients with more than one of: chronic otalgia, bloody otorrhoea, bleeding, mass, facial swelling or palsy should be biopsied (R)
• Magnetic resonance and CT imaging should be performed (R)
• Patients should undergo audiological assessment (R)
• Carotid angiography is recommended in select patients (G)
• The modified Pittsburg T-staging system is recommended (G)

### Treatment planning and prognosis

There should be a specific multidisciplinary team (MDT) dealing with skull base cancers. For sarcomas, there should be liaison with the sarcoma MDT, and, for paediatric sarcomas, with the paediatric oncology MDT.

Most patients with operable cancer of the lateral skull base are treated with primary surgery, with the exception of some sarcomas. Given the low incidence of lateral skull base cancer, the variety of precise sites of origin, heterogeneity of tumour pathology and individual circumstance, it is difficult to generalise treatment guidelines. The commonest scenarios are of SCC arising in the ear or TB (ME and/or EAM) and advanced parotid cancers. The situation of advanced cutaneous SCC invading the TB is not materially different.^3^

## Cancers arising in the temporal bone

### General principles

For cancer arising in the TB, the most favourable survival rates are achieved with an en bloc extended TB resection and post-operative radiotherapy (RT).^4^^–^^6^ The influence of ME involvement on prognosis is critical. T1 and T2 lesions lateral to the tympanic membrane have cure rates near to 100 per cent with true enbloc resections without breach of the tumour. The majority of T3 tumours are also cured with disease specific survival rates over 70 per cent, whereas T4 five-year survival results vary between 30 and 50 per cent.^1^^,^^7^^,^^8^ Nodal metastasis has a major influence on prognosis.^5^^,^^9^ Equally critical for prognosis is a histologically-proven complete microscopic resection.^4^^,^^8^

Extension superiorly through the tegmen leads to dural and cerebral involvement. Dural involvement is an adverse prognostic indicator, but around one-third of such patients are curable with the appropriate surgery.^5^ Cerebral involvement rarely confers any chance of cure.

On the other hand, T4 tumours that are T4 by virtue of anterior invasion to the TMJ and/or pre-auricular tissue have a much better prognosis than other T4 tumours.^9^

Resection of the intra-petrous carotid is possible. Some patients can benefit from pre-operative radiological permanent occlusion of the carotid artery, subject to successful balloon occlusion. However, the cancer-mortality in this group of patients with petrous apex involvement is high, due to difficulties achieving full microscopic resection around this area, and the post-operative morbidity is high due to, amongst other things, multiple cranial nerve deficits from a resection of this extent.

Thus, for patients with a combination of high morbidity with low chance of surgical cure, consideration should be given not to offer primary surgery.^5^

### Temporal bone surgery

#### Lateral temporal bone resection (LTBR) should be regarded as the minimum oncological operation for T1 and T2 lesions^5^^,^^10^

Essential elements of LTBR are (1) excision lateral to facial nerve; (2) conchal bowl resection; and (3) bony cuts: mastoid to middle fossa dura (or leaving a thin layer of bone), anteriorly into zygomatic aircells and TMJ, inferiorly to stylomastoid foramen, hypotympanum to TMJ.

Additional options include (see below): resection of entire pinna and periauricular skin; condyle/mandible, parotid, extension of resection into parapharyngeal space and infratemporal fossa, neck dissection, facial nerve sacrifice and cable graft.

#### Extended temporal bone resection (ETBR) is required for more extensive tumours involving the middle ear^11^

The essential elements of EBTR are (1) facial nerve sacrifice; (2) posterior and middle craniotomy; (3) labyrinthectomy; (4) transection of internal auditory canal; (5) resection of petrous tip; (6) exposure of intra-petrous portion of the carotid; and (7) total parotidectomy.

Additional options include: craniectomy (squamous TB; sphenoid wing, posterior fossa); mandibulectomy; parapharyngeal and/or infratemporal fossa resection; extension to jugular foramen; lower cranial nerve sacrifice; internal carotid artery; dura; brain.

**Recommendation**

• The minimum operation for cancer involving the temporal bone is a lateral temporal bone resection (R)

### Resection of other structures in TB surgery

#### Parotid gland

When performing TB resections for TB cancers and advanced skin cancers, the parotid gland may be either involved directly by tumour or be harbouring intra-parotid lymph node metastases (it may contain the primary echelon lymph node). The former may be suggested by pre-operative scans. Therefore, for all resections, at least a superficial parotidectomy should be carried out.^10^ For advanced T3/T4 TB SCCs, total parotidectomy should be carried out, which also facilitates access to the parapharyngeal space, infratemporal fossa and masticator space. For basal cell carcinoma (BCC) without evidence of direct invasion into or near the parotid gland, parotidectomy can be omitted.

#### Temporo-mandibular joint/mandible

The standard anterior bony cut in a lateral TB resection goes into the TMJ. There is therefore some degree of disruption of TMJ function as a consequence. If there is involvement of or near the TMJ/condyle, it is recommended that a partial mandibulectomy is carried out, which may range from condylectomy to resection from mandibular notch to angle. If the latter is done, the inferior alveolar nerve should be preserved, if oncologically sound to do so. There is, however, no need for routine resection to include the TMJ in lateral temporal bone resection (LTBR).^5^

## Temporal bone resection in parotid cancers

Almost all parotid cancers abutting the TB are easier to remove if an inferior TB resection is done to get medial and posterior to the tumour rather than finding the facial nerve outside the stylomastoid foramen and getting too close to the tumour. This improvement in surgical access both improves prognosis and ease of facial nerve grafting if required.^12^^,^^13^ For parotid tumours with EAM or TB involvement, at least a lateral TB resection will be required.

## Facial nerve

Facial nerve involvement by tumour is a significant adverse prognostic factor. Pre-operative facial nerve dysfunction due to facial nerve involvement by tumour requires sacrifice of the nerve as part of the resection required. For some patients with normal function pre-operatively, it may be technically impossible to resect a tumour without nerve sacrifice if the nerve is totally encased by tumour, bearing in mind the aim of surgery is complete, preferably monobloc, tumour resection with margins. When the facial nerve is sacrificed, the proximal stump at the limit of the sacrifice should be sent for frozen section pathology.

In cases in which nerve sacrifice is necessary, one or more of the following steps should be considered detailed below. It should be borne in mind that the best time to perform many of these interventions is at the time of tumour resection, as virtually every patient in this group will go on to have post-operative RT.

A cable graft from ME facial nerve to intra-parotid branches can be performed if (a) there is enough proven tumour-free proximal facial nerve (otherwise a facial-hypoglossal anastamosis can be considered) and (b) if the peripheral branches can be identified (this may be difficult when a radical en-bloc parotidectomy with overlying skin is performed). Useful donor nerves include greater auricular nerve, sural nerve or lateral cutaneous nerve of thigh (easily available if harvesting an anterolateral thigh free flap). If an alternative lengthening of telomeres (ALT) free flap is to be employed, this can be used as a chimaeric flap, with separate components for volume restoration and facial function and vascularised interposition nerve grafting.

Otherwise, either static procedures can be employed such as fascia lata sling for oral commissure/cheek suspension or dynamic procedures such as lengthened temporalis myoplasty (e.g. Labbé type I or II), if the deep temporal nerve and artery are preserved. Oculoplastic interventions (e.g. gold weight, canthoplasty) can be performed at the time of tumour resection or later on.

**Recommendation**

• Facial nerve rehabilitation should be initiated at primary surgery (G)

## Reconstruction

The aims of reconstruction of lateral skull base defects can be considered hierarchically:

• Protection for the brain when the dura mater is breached.
• Skin defect.
• Auricular defect.
• Tissue volume defect and mandible defect.
• Functional defect-facial nerve.

Dural defects are normally repaired with non-vascularised tissue such as autologous fascia lata grafts, pericardial xenografts or synthetic materials.

Reconstruction of the skin defect should be considered with the volume defect, this being determined by extent of temporal bone resection, parotidectomy and mandibulectomy in particular.^14^

For smaller skin defects without much volume loss, options include radial forearm free flap, cervicofacial rotation flap, temporalis flap and supraclavicular artery island flap. These can be used to reconstruct small skin/auricle defects with modest volume loss.

For most defects after temporal bone resection, the anterolateral thigh free flap offers optimal reconstruction, offering bulk (variable by the inclusion of vastus lateralis), and enough skin for most defects (which can be reduced by de-epithelialisation if the auricle is not resected).^14^ It is reliable, has the requisite tissue and minimal donor site morbidity. It allows vascularised fascia lata to be used for static facial resuspension or the lateral cutaneous femoral nerve for either sensory innervation of the flap or an interpositional facial nerve graft. Also, the accessible donor site allows for concomitant flap harvest and tumour ablation. Alternative flaps include latissimus dorsi, rectus abdominis or deep inferior epigastric artery perforator, radial forearm, medial sural artery and lateral arm flaps.

In a vessel-depleted neck or in a patient unsuitable for microvascular surgery, lower trapezius muscle island flap (if the transverse cervical vessels are intact) or superior trapezius flap (when a radical neck dissection has been performed) can be used. The use of pectoralis major or delto-pectoral flap is sub-optimal as the lateral skull base is at or beyond the limits of rotation in many cases.

It is feasible to leave selected condylar resections unreconstructed accepting minor dental occlusal disturbance. Where mandibular reconstruction is required, a composite microvascular flap such as a chimeric thoracodorsal artery perforator – scapular osteomusculocutaneous flap can restore a large mandibular and lateral skull defect.

**Recommendation**

• Anterolateral thigh free flap is the workhorse flap for lateral skull base defect reconstruction (G)

## Neck dissection

Up to 20 per cent of patients with temporal bone SCC will have lymph node metastases. The need for neck dissection depends on the pathology. As for any head and neck cancer, clinically or radiologically staged N + necks require comprehensive neck dissection, but level 1a (submental) can be spared. In the setting of N0 neck, it is also recommended that neck dissection (levels 1b, 2–5) is performed for all temporal bone SCC.^15^ The same applies to advanced parotid carcinomas with temporal bone involvement.

**Recommendation**

• For patients undergoing surgery for squamous cell carcinoma, at least a superficial parotidectomy and selective neck dissection should be carried out (R)

## Radiation therapy

### Post-operative RT

Most T2–T4 SCCs will require post-operative RT,^5^ as will advanced parotid cancers requiring temporal bone surgery. T1 and selected T2 SCCs without adverse histological features (particularly peri-neural infiltration) and with proven clear margins may not require adjuvant therapy. Dosimetry with electrons is unpredictable due to tissue heterogeneity and photon therapy is preferred using three-dimensional conformal or intensity modulated techniques (IMRT). The clinical target volume is determined from pre-operative imaging and further informed from MDT feedback on operative and histopathological findings.

Conformal RT is computer planned and the target volume often resembles a transaxial triangular shape with the base laterally. A simple pair of horizontal wedged lateral oblique fields may suffice, with beams exiting on either side of the contralateral parotid. An additional lateral field with vertical wedging may improve homogeneity longitudinally.

Intensity modulated techniques may well reduce dose to the ipsilateral cochlea (if this is separate from the tumour volume) and oral cavity. Chronic otomastoiditis and TB necrosis following RT can be reduced by restricting the volume of bone treated to high dose as far as possible. The contralateral parotid, bilateral submandibular glands, oral cavity, mandible, cochlea as well as central nervous system (CNS) structures should be routinely contoured and given constraint doses.

Post-operative doses used for head and neck cancer are 60 Gy in 30 fractions for moderate risk and 66 Gy in 33 fractions for high risk; these doses can potentially be applied for lateral skull base cancers, but the normal tissue (particularly CNS) complication rate is clinically significant at doses above 60 Gy. Synchronous post-operative treatment with cisplatin can be also considered.^16^

### Primary RT

When primary surgery is not considered possible, or too morbid, definitive RT may be used, with overall cure rates of just under half of patients overall.^16^ Clinical target volume is based on staging imaging, preferably with both CT and MR imaging (MRI). Higher biological doses are used compared with the post-operative setting so that optimal conformality is essential to reduce treatment complications. Standard IMRT doses can be used: 66 Gy in 30 fractions for macroscopic disease, 60 Gy for high risk microscopic areas and 54 Gy for moderate risk microscopic areas; these doses may be modified according to the volume of CNS tissue in the clinical target volume. In view of the emphasis on conformality, there may well be a role for proton beam therapy in some cases.

Synchronous treatment with cisplatin can be considered; an alternative strategy is to use cetuximab.

## Other lateral skull base cancer operations

Tumours of the infratemporal fossa are more rare and heterogeneous and thus need an individualised operative approach. Examples include facial translocation, sub-temporal pre-auricular, orbito-zygomatic and trans-TB (Fisch) approaches.^17^^–^^20^

## Post-operative care issues

In addition to VII nerve issues, all lower cranial nerves essential for swallowing and voice (IX, X, XII) are at risk of injury or sacrifice in surgery for advanced tumours. Care of the patient in this situation must include close involvement of speech and language therapy. Interventions include either pre- or post-operative percutaneous gastrostomy; naso-gastric tube; tracheostomy if aspirating on saliva. Later interventions include vocal cord medialisation and crico-pharyngeal myotomy.

Ipsilateral total or total conductive hearing deficit is an inevitable outcome of TB resection. Pre-operative audiological assessment of the contralateral ear will identify patients with a pre-existing deficit. This may be corrected or improved with appropriate aiding in either the pre- or post-operative period. Total conductive hearing loss can be rehabilitated through an osseo-integrated bone anchored hearing aid (BAHA). Total hearing loss can be rehabilitated through either a BAHA or a bilateral contralateral routing of signals (BI-CROS) aid.

Post-operative vertigo is expected if there is resection of a functioning labyrinth. If vestibular compensation is protracted and incomplete, referral for vestibular rehabilitation services should be considered.

## Palliative care

The local issues that affect patients when tumours are inoperable or recur are generally pain (particularly through dural involvement) and fungation. Therefore, the instigation of a comprehensive analgesic regimen is required. Fungation can be a particular problem, made worse by the prominent site of the cancer. Radiotherapy can be given for palliative intent, if not already given, and can be useful for both pain and fungation. Short fractionation schedules may well be appropriate in these situations using, for example, 30 Gy in 10 fractions and a single lateral megavoltage photon field. If RT has previously been given and there is a reasonable interval (more than 12 months), then re-irradiation is sometimes beneficial.

### Key points

• Cancer of the lateral skull base is rare and constitutes a heterogeneous group of cancers and sites of origin
• Most cancers are treated with primary surgery and post-operative radiotherapy
• For temporal bone cancers, the boundary of the tympanic membrane is paramount in prognosis. Most T1 and 2, and many T3 cancers are cured
• The minimum operation for a temporal bone cancer should be a lateral temporal bone resection
• Lateral temporal bone resection should be considered in advanced parotid cancers
• Achieving clear microscopic margins at surgery is critical
• Salvage surgery is often not successful: the best, and usually only, chance of cure is at initial surgery
• For patients with advanced cancers, particularly at the petrous apex or with dural or facial nerve involvement, cure rates drop considerably
• For patients with advanced cancers undergoing surgery, there are many rehabilitation issues
• The anterolateral thigh free flap is the workhorse for reconstruction.

**Table II:** Modified Pittsburg staging system^2^

T1	Tumour limited to the EAC without bony erosion or evidence of soft tissue extension
T2	Tumour with limited EAC erosion (not full thickness) or radiological findings consistent with limited (<0.5 cm) soft tissue involvement
T3	Tumour eroding the osseous EAC (full thickness) with limited (<0.5 cm) soft tissue involvement of middle ear and/or mastoid or causing facial paralysis at presentation
T4	Tumour eroding the cochlear, petrous apex, medial wall of middle ear, carotid canal, jugular foramen, or dura or with extensive (>0.5 cm) soft tissue involvement

## References

[1] Leong SC, Youssef A, Lesser TH. Squamous cell carcinoma of the temporal bone: outcomes of radical surgery and postoperative radiotherapy. Laryngoscope 2013;123:2442–48 23553471 10.1002/lary.24063

[2] Moody SA, Hirsch BE, Myers EN. Squamous cell carcinoma of the external auditory canal: an evaluation of a staging system. Am J Otol 2000;21:582–88 10912706

[3] Essig GF, Kitipornchai L, Adams F, Zarate D, Gandhi M, Porceddu S Lateral temporal bone resection in advanced cutaneous squamous cell carcinoma: report of 35 patients. J Neurol Surg B, Skull Base 2013;74:54–9 24436889 10.1055/s-0032-1331021 PMC3699167

[4] Bacciu A, Clemente IA, Piccirillo E, Ferrari S, Sanna M. Guidelines for treating temporal bone carcinoma based on long-term outcomes. Otol Neurotol 2013;34:898–907 23507994 10.1097/MAO.0b013e318281e0a9

[5] Masterson L, Rouhani M, Donnelly NP, Tysome JR, Patel P, Jefferies SJ Squamous cell carcinoma of the temporal bone: clinical outcomes from radical surgery and postoperative radiotherapy. Otol Neurotol 2014;35:501–8 24492135 10.1097/MAO.0000000000000265

[6] Madsen AR, Gundgaard MG, Hoff CM, Maare C, Holmboe P, Knap M Cancer of the external auditory canal and middle ear in Denmark from 1992 to 2001. Head Neck 2008;30:1332–8 18704969 10.1002/hed.20877

[7] Lassig AA, Spector ME, Soliman S, El-Kashlan HK. Squamous cell carcinoma involving the temporal bone: lateral temporal bone resection as primary intervention. Otol Neurotol 2013;34:141–50 23202152 10.1097/MAO.0b013e318278bf38

[8] Yin M, Ishikawa K, Honda K Analysis of 95 cases of squamous cell carcinoma of the external and middle ear. Auris Nasus Larynx 2006;33:251–57 16431060 10.1016/j.anl.2005.11.012

[9] Mazzoni A, Danesi G, Zanoletti E. Primary squamous cell carcinoma of the external auditory canal: surgical treatment and long-term outcomes. Acta Otorhinolaryngol Ital 2014;34:129–37 24843224 PMC4025180

[10] Zhang T, Li W, Dai C, Chi F, Wang S, Wang Z. Evidence-based surgical management of T1 or T2 temporal bone malignancies. Laryngoscope 2013;123:244–8 23008138 10.1002/lary.23637

[11] Chang CH, Shu MT, Lee JC, Leu YS, Chen YC, Lee KS. Treatments and outcomes of malignant tumors of external auditory canal. Am J Otolaryngol 2009;30:44–8 19027512 10.1016/j.amjoto.2008.02.007

[12] Mehra S, Morris LG, Shah J, Bilsky M, Selesnick S, Kraus DH. Outcomes of temporal bone resection for locally advanced parotid cancer. Skull Base 2011;21:389–96 22547966 10.1055/s-0031-1287682 PMC3312127

[13] Shao A, Wong DK, McIvor NP, Mylnarek AM, Chaplin JM, Izzard ME Parotid metastatic disease from cutaneous squamous cell carcinoma: prognostic role of facial nerve sacrifice, lateral temporal bone resection, immune status and P-stage. Head Neck 2014;36:545–50 23780509 10.1002/hed.23323

[14] Hanasono MM, Silva AK, Yu P, Skoracki RJ, Sturgis EM, Gidley PW. Comprehensive management of temporal bone defects after oncologic resection. Laryngoscope 2012;122:2663–9 23070792 10.1002/lary.23528

[15] Rinaldo A, Ferlito A, Suarez C, Kowalski LP. Nodal disease in temporal bone squamous carcinoma. Acta Otolaryngol 2005;125:5–8 15799566 10.1080/00016480410018287

[16] Takenaka Y, Cho H, Nakahara S, Yamamoto Y, Yasui T, Inohara H. Chemoradiation therapy for squamous cell carcinoma of the external auditory canal: a meta-analysis. Head Neck 2015;37:1073–80 24692266 10.1002/hed.23698

[17] Sekhar LN, Schramm VL Jr, Jones NF. Subtemporal-preauricular infratemporal fossa approach to large lateral and posterior cranial base neoplasms. J Neurosurg 1987;67:488–99 3655886 10.3171/jns.1987.67.4.0488

[18] Nuss DW, Janecka IP, Sekhar LN, Sen CN. Craniofacial disassembly in the management of skull-base tumors. Otolaryngol Clin North Am 1991;24:1465–97 1792080

[19] Suarez C, Llorente JL, Munoz C, Garcia LA, Rodrigo JP. Facial translocation approach in the management of central skull base and infratemporal tumors. Laryngoscope 2004;114:1047–51 15179211 10.1097/00005537-200406000-00017

[20] Timoshenko AP, Asanau A, Gavid M, Colin V, Martin C, Prades JM. Preauricular transmandibular and transzygomatic approach for tumors of the infratemporal fossa revisited. ORL J Otorhinolaryngol Relat Spec 2013;75:250–5 23921824 10.1159/000351554

